# Supplementary material for: Grenadian cats as potential reservoir for Leptospira
Source: PLoS Negl Trop Dis. 2024 Dec 26;18(12):e0012784. doi: 10.1371/journal.pntd.0012784 (PMC11709269; doi:10.1371/journal.pntd.0012784)
Supplement: Attachment — Submitted filename: PNTD-D-24-00107_reviewer_2024v1 REPORT1.pdf [file pntd.0012784.s003.pdf]

Dear Authors and Editors,

Thanks for giving me the opportunity to review this interesting manuscript.

This study describe the possibility that Grenadian cat population may be a reservoir host for zoonotic Leptospira on the island and can be a source of leptospirosis.

General comments: The study aims to characterize the circulation of *Leptospira* among stray cats in a selected area of Grenada. The animals sampled were very young, which may have resulted in a shorter period of exposure to the pathogen than the average cat's life. Additionally, a serological screening test was used that is not validated for use in cats. When comparing the prevalence of the pathogen in different countries, it is important to consider the diagnostic methods used and other relevant variables. In the following paragraph, I have provided some suggestions and would appreciate a prompt response. If there are any insurmountable obstacles, a 'study limitations' paragraph must be included.

It is also necessary to contextualize the statements by quoting bibliographical references and appropriate citations. Additionally, please ensure correct grammar, punctuation, and proper formatting when indicating the names of the genera and species of microorganisms. Please check all the document for using the correct form (italics) in idicating the genus of the bacteria. Although the writer is fluent in English, revisions are required for form, grammar, and typos.

Overall recomendation: Maior revision required

Abstract
Please check for typos. In addition, you state you had performed the molecular analysis on serum samples.. Is that correct?

Introduction
Line 118- …: I would suggest contextualising the role of the cat as a possible **reservoir** or as an asymptomatic **carrier** (difference in epidemiological role). What may be the source of exposure (predatory habits, local wildlife and synanthropy, stray and wild animal population density)?

M&M and Results:
Could selecting young animals (median age 1 y/o) be a limitation of the study? The exposure time to the pathogen may not be representative of the general stray cat population. How can you address this bias?
Line 167: please also state the drugs commercial name, manufacturer and country, as per author guidelines.
Line 197: Which modification?
Line 229: The assessment of serological positivity in cats performed with an antibody immunochromatographic test for dogs could produce inaccurate results. Is it possible that sera could be tested with a more sensitive and specific method (MAT)?

Discussion:
Lines 310-…: Please discuss your results by contextualizing the techniques used by previous studies, both in terms of molecular and serological techniques.
Line 339-340: Disease stage and *Leptospira* elimination detected by PCR, should be discussed with a view to characterizing a possible carrier or reservoir
Are there any published studies on *Leptospira* positivity in bats in Grenada? Are there any ecological studies that justify scenario 2? the bibliography needs to be implemented.
It is not clear to me whether the cats were owned (so informed consent from the owner was required) or were strays/feral.
A legend for the size of the population of cats enrolled in the study must be added to Figure 1.
