# Supplementary material for: Grenadian cats as potential reservoir for Leptospira
Source: PLoS Negl Trop Dis. 2024 Dec 26;18(12):e0012784. doi: 10.1371/journal.pntd.0012784 (PMC11709269; doi:10.1371/journal.pntd.0012784)
Supplement: Attachment — Submitted filename: Review response letter.pdf [file pntd.0012784.s005.pdf]

St. George's University

September 30, 2024

Dear editor,

Once again, we would like to sincerely thank the reviewers and the editorial board for reviewing our manuscript and for giving us the opportunity to respond to the queries raised. We have considered all the review quires and have responded to them. Please find below responses to the reviews.

The authors would like to thank **reviewer number #1** for the review considerations of the manuscript.

Reviewer #2: -Are the objectives of the study clearly articulated with a clear testable hypothesis stated? No.

Response: As shown by several studies, cats infected with *Leptospira* can be asymptomatic. Furthermore, it has also been shown that infected cats are able to shed *Leptospira* in their urine even after clearance of infection (Levett PN 2001 and other authors).

In Grenada, *Leptospira* in cats (domestic and feral) is not well studied, hence this study aimed to determine if Grenadian feral cats are infected with *Leptospira.*

-Is the study design appropriate to address the stated objectives?

Response: Based on the PCR findings in the cat's urine examined in this study, it is observed that Grenadian feral cats even though were asymptomatic are infected with *Leptospira*. However, the window of shedding could not be established in the current study. This is interesting point which needs to be considered in the future studies.

-Is the population clearly described and appropriate for the hypothesis being tested?

Response: The authors agree that many of the cats selected were very young and may have not had exposure to the pathogen. This is a limitation which the authors have acknowledged in the limitation of the study (please refer to line 373-390).

-Is the sample size sufficient to ensure adequate power to address the hypothesis being tested?

-Were correct statistical analysis used to support conclusions?

Response: Yes, the correct statistical analysis were used to support the study findings (See line 253-254 and lines 335-336).

With regarding the sample size and prevalence determination, please refer to lines 162-165 of the manuscript. The total 12% prevalence was based on the fact that Grenadian cats would be exposed infected and shedding with a 5% margin of error and 95% confidence level. From the Caribbean region, similar prevalence's were observed in Trinidad and St Kitts (lines 303-304).

-Are there concerns about ethical or regulatory requirements being met?

School of Veterinary Medicine

University Centre, Grenada, West Indies ✦ Phone: 473-439-2000 ✦ Fax: 473-444-1480

St. George's University

Response: The study was conducted according to the guidelines approved by the Institutional Animal Care and Use committee of St. George's University (IACUC #18016-R), these regulatory requirements are stated in the materials and methods section of the manuscript.

DNA extraction and PCR analysis: The description of the extraction methods, processing of *Leptospira* DNA from serum is inadequate. in addition, it is reported that unsuitable extraction kits (VIRAL) were used, and the result of the serum PCR is not even described.

Response: The extraction of DNA from urine and fecal samples can be challenging due to degradation of and PCR inhibitors. To overcome these challenges, the authors used the Viral Qiagen Kit to extract DNA from urine samples. This procedure is not new, it has been used previously by other researchers in the past (Chan et al). This kit (protocol) is able inactivate the PCR inhibitors present in urine sample. We have updated this protocol in the manuscript (See line 204-212). On the other hand, the serum PCR results are presented in the results section (Please see 253-255).

Serology/other tests

Response: We agree with the reviewer that a comparative test including MAT would have been an additional test, however, in the current study the amount of blood sample collected from the cats was limited to comply with IAACUC requirements. As a result, we did not have enough sample to perform additional tests as part of this study. To address this limitation, future studies should include a larger sample size and from all parishes in Grenada to get a better representation of the feral cat population on the island. This would likely expand the age range.

I suggest removing this part or contextualizing the use of serum by combining two serological methods (ELISA) and a gold standard method (MAT)

Response:

Reviewer #2: -Are the conclusions supported by the data presented? yes -Are the limitations of analysis clearly described? yes -Do the authors discuss how these data can be helpful to advance our understanding of the topic under study? yes -Is public health relevance addressed? Yes

The authors acknowledge the comments and suggestions made by the reviewers. As both reviewers agree that the results, conclusions, and limitation are adequately established, the authors would like to request that findings of the study be presented as such.

Kindly find attached, the tracked changes of the manuscript and a clean copy. We hope the responses along with the revised manuscript will be considered for publication.

Thank you very much.

Andy Alhassan

School of Veterinary Medicine

University Centre, Grenada, West Indies ♦ Phone: 473-439-2000 ♦ Fax: 473-444-1480
