# Supplementary material for: Grenadian cats as potential reservoir for Leptospira
Source: PLoS Negl Trop Dis. 2024 Dec 26;18(12):e0012784. doi: 10.1371/journal.pntd.0012784 (PMC11709269; doi:10.1371/journal.pntd.0012784)
Supplement: Attachment — Submitted filename: PNTD-D-24-00107_reviewer_2024v2 REPORT - Copia.docx.pdf [file pntd.0012784.s006.pdf]

Dear Authors and Editors,

I am grateful for the chance to revise the manuscript once more.

The manuscript has been revised and improved. A few comments and suggestions for further enhancements remain.

Overall recomendation: Minor revision required

Introduction

Line 110: Please add more updated bibliography (2, 18, 20, 21, 22, ..)

Line 118- "Hence cats serve as a source of infection and a environmental sentinel for Leptospira (20)."

Line 128: typing errors

M&M and Results:

Line 240: Please specify which values you considered statistically significant. Please indicate the technic characteristics of the statistic program used.

Conclusion:

Line 359: It would appear that there is no evidence to suggest that the cat in question poses a direct risk of infection to humans. Indeed, recent literature would seem to indicate that with regard to pathogenic leptospires (see list in various publications), companion animals (dogs, cats in particular) may act as epidemiological sentinels, given that the disease is considered to be an environmental disease. This sentence must be revised ("It is important to educate the public about the potential risk of exposure to *Leptospira* via feral cats.")
