# Supplementary material for: Grenadian cats as potential reservoir for Leptospira
Source: PLoS Negl Trop Dis. 2024 Dec 26;18(12):e0012784. doi: 10.1371/journal.pntd.0012784 (PMC11709269; doi:10.1371/journal.pntd.0012784)
Supplement: Attachment — Submitted filename: Letter-PNTD-D-24-00107R2.pdf [file pntd.0012784.s007.pdf]

St. George's University

November 27, 2024

Dear editor,

Once again, we would like to sincerely thank the reviewers and the editorial board for reviewing our manuscript. We have considered all the review quires and suggestions and have responded accordingly.

Please find below responses to the reviews.

Reviewer #2:

Line 110: Please add more updated bibliography (2, 18, 20, 21, 22, ..)

Response: The references numbers (bibliography) has been updated in the entire document.

Line 118- "Hence cats serve as a source of infection and a environmental sentinel for Leptospira (20)."

Response: Hence cats serve as a source of infection and a sentinel for *Leptospira* (see page 6, lines 115-116 of the clean revised manuscript).

Line 128: typing errors

Response: word flequesntly has be corrected to "frequently" (see page 6, line 128 of the clean revised manuscript).

M&M and Results:

Line 240: Please specify which values you considered statistically significant. Please indicate the technic characteristics of the statistic program used.

Response: A P value of < 0.05 is considered statistically significant while a P value >0.05 is statistically insignificant (69). (see page 12, lines 242-243 of the clean revised manuscript).

Conclusion: Line 359: It would appear that there is no evidence to suggest that the cat in question poses a direct risk of infection to humans. Indeed, recent literature would seem to indicate that with regard to pathogenic leptospiral (see list in various publications), companion animals (dogs, cats in particular) may act as epidemiological sentinels, given that the disease is considered to be an environmental disease. This sentence must be revised ("It is important to educate the public about the potential risk of exposure to Leptospira via feral cats.")

Response: We have revised the conclusion section to read as follows: (see page 19, lines 365-373 of the clean revised manuscript).

School of Veterinary Medicine

University Centre, Grenada, West Indies ♣ Phone: 473-439-2000 ♣ Fax: 473-444-1480

St. George's University

Although the exact role that cats play in the epidemiology of human leptospirosis is unclear, it is important to recognize the potential risk of exposure to *Leptospira* via feral cats to both human and their pets. Results of this study can help inform the Grenada community about the potential risk of exposure through contaminated water, soil and infected urine. Furthermore, it is important for veterinary practitioners to be aware that cats may present with *Leptospira* infection and to consider leptospirosis as a differential in feline patients. Importantly, the results of this study and other studies confirm pathogenic and zoonotic *Leptospira* in cats. Vaccinating cats against *Leptospira* needs to be considered and potentially become part of core feline vaccines in endemic areas to minimize cat infections and shedding of *Leptospira* with potential human exposure.

Kindly find attached, the tracked changes of the manuscript and a clean copy for your consideration.

We hope the responses along with the revised manuscript will be considered for publication.

Thank you very much.

Andy Alhassan

School of Veterinary Medicine

University Centre, Grenada, West Indies ✦ Phone: 473-439-2000 ✦ Fax: 473-444-1480
