# Supplementary material for: Grenadian cats as potential reservoir for Leptospira
Source: PLoS Negl Trop Dis. 2024 Dec 26;18(12):e0012784. doi: 10.1371/journal.pntd.0012784 (PMC11709269; doi:10.1371/journal.pntd.0012784)
Supplement: Attachment — Submitted filename: Review Responses-Kalasi et al 2024.docx [file pntd.0012784.s004.docx]

**Review Responses**

Responses to reviewer #1

**Comment**: **line 32:** I recommend changing the word "confirm" to "suggest"

**Response**: the word confirm has been changed to **suggest**.

**Comment**: **line 112:** I recommend starting a new sentence with the word "Furthermore"

**Response**: we have added the word **furthermore**

**Comment:** line 201: I recommend changing the word "Then" to "Next" to start the sentence

**Response**: the word then has changed to **next**.

**Comment:** line 213: reference (62) needs to be unbold

line 299: " La Scola et al. (63) need to be unbold

**Response**: the references have been **unbold**.

**Comment:** line 352: I recommend changing the word "Lepto" to "Leptospira" and have it italicized

**Response**: Lepto has been changed to ***Leptospira*** and italicized.

**Comment:** line 353: the dot (.) after "Furthermore," needs to be removed

**Response**: the (.) has been removed.

Responses to reviewer #2

The typos in the abstract and the entire manuscript has been revised as recommended (please see tracked changes in the original document).

**Comment**: In addition, you state you had performed the molecular analysis on serum samples... Is that correct?

**Response**: Yes, molecular analysis was performed on the serum samples. We have included additional information of the molecular analysis in the materials and methods section. Please see the **(page 12, line 232)** in the revised manuscript.

**Comment**:Introduction
Line 118- ..: I would suggest contextualising the role of the cat as a possible reservoir or as an asymptomatic carrier (difference in epidemiological role). What may be the source of exposure (predatory habits, local wildlife and synanthropy, stray and wild animal population density)?

**Response**: We appreciate this comment. Indeed, the source of exposure of cats to *Leptospira* as reported by other researchers might be due to the fact that stray cats come into contact with the *Leptospira* rodent reservoir hosts, perhaps through hunting habits. We have incorporated this information in the revised manuscript **(please see page 6, line 128-130)**

**Comment**: M&M and Results:
Could selecting young animals (median age 1 y/o) be a limitation of the study? The exposure time to the pathogen may not be representative of the general stray cat population. How can you address this bias?

**Response**: Yes, we agree with the reviewers comment regarding the study population. This is a limitation which we have addressed and included in the revised manuscript **(page 24, line 423-439)**

**Comment**: Line 167: please also state the drugs commercial name, manufacturer and country, as per author guidelines.

**Response**: The drugs commercial name, manufacturer and country have been incorporated into the revised manuscript (**please see page 10, line 187-190)**

**Comment:** Line 197: Which modification?

**Response:** the sentence with modification has been removed since there wasn’t any significant modification to the protocol.

**Comment**: Line 229: The assessment of serological positivity in cats performed with an antibody immunochromatographic test for dogs could produce inaccurate results. Is it possible that sera could be tested with a more sensitive and specific method (MAT)?

**Response:** We agree with the reviewer’s assessment of this serological method used which could have resulted in inaccurate results. We have removed this serological assessment from the entire study.
